# Hypertensive Emergency Secondary to Paraganglioma in a Pediatric Patient With a Fenestrated Fontan

**DOI:** 10.1016/j.jaccas.2025.104934

**Published:** 2025-08-06

**Authors:** Kara Gay-Simon, Kathryn Forbes, Jose Ugarriza Mendoza, Dyana Conway, Luisa F. Angel, Jason Katz, Abdulwahab Aldousany

**Affiliations:** a Department of Cardiology, Cardiac Critical Care Medicine at Nicklaus Children's Hospital, Miami, Florida, USA; b Department of Cardiac Critical Care Medicine at Nicklaus Children's Hospital, Miami, Florida, USA; c Kidz Medical Services, Coral Gables, Florida, USA

**Keywords:** Fontan, hypertension, hypoxia, paraganglioma, pediatric, pheochromocytoma

## Abstract

**Background:**

Children with Fontan physiology are at risk for a range of complications, including potentially life-threatening causes of secondary hypertension.

**Case Summary:**

We present a 10-year-old girl with hypoplastic left heart syndrome post-Fontan who developed hypertensive crisis, chest pain, and atrial tachycardia. Work-up revealed elevated cardiac biomarkers and a retroperitoneal mass at the aortic bifurcation. Laboratory testing showed markedly elevated catecholamines, and imaging confirmed a catecholamine-secreting paraganglioma. She was medically stabilized with alpha and beta blockade, and then underwent successful surgical resection. She was discharged with well-controlled blood pressure and continues to receive multidisciplinary follow-up.

**Discussion:**

Paragangliomas are rare in children but may be linked to chronic hypoxia in congenital heart disease. In Fontan patients, sustained hypoxemia may predispose to neuroendocrine tumors, necessitating vigilance for secondary causes of hypertension.

**Take-Home Message:**

Early recognition and coordinated care are essential in managing secondary hypertension in children with complex congenital heart disease.

## Graphical Abstract

**Figure:**
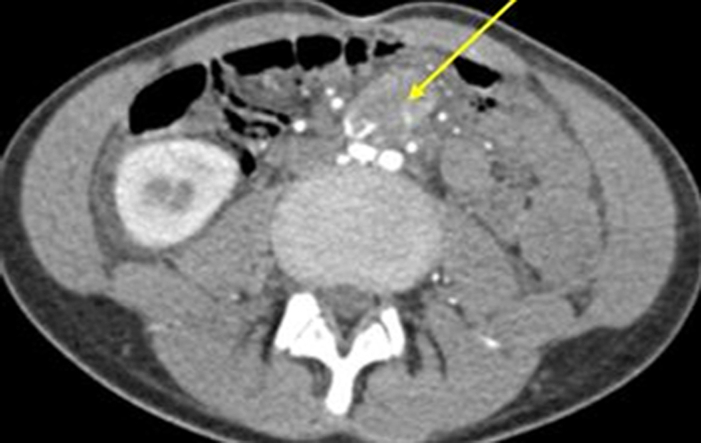

A 10-year-old girl with a history of hypoplastic left heart syndrome and Fontan physiology presented with acute hypertension (200/120 mm Hg), intermittent chest pain, and new-onset atrial tachycardia. Her examination revealed tachycardia and a systolic murmur; oxygen saturation was 94% on room air.

**Take-Home Messages**

• Paraganglioma should be considered in the differential diagnosis of pediatric hypertension, particularly in patients with cyanotic congenital heart disease and Fontan physiology.
• Early recognition and multidisciplinary perioperative management are essential for safe resection and favorable outcomes in catecholamine-secreting tumors.

Laboratory evaluation demonstrated elevated cardiac biomarkers (troponin I: 0.041 ng/mL; troponin T: 25 ng/L), a markedly elevated pro–B-type natriuretic peptide (7110 pg/mL), and signs of hemoconcentration (hemoglobin: 17.6 g/dL, hematocrit: 51.3%). *Mycoplasma pneumoniae* immunoglobulin M was reactive.

Initial blood pressure management with intravenous hydralazine was ineffective. Nicardipine infusion reduced blood pressure but induced narrow-complex tachycardia (180-205 beats/min). Lack of response to adenosine suggested nonreentrant atrial tachycardia and was thought to represent ectopic atrial tachycardia. Esmolol was initiated, and nicardipine was discontinued. She was transferred for tertiary care.

## Past Medical History

Hypoplastic left heart syndrome status post extracardiac fenestrated Fontan at 4 years of age.

## Differential Diagnosis

Causes of secondary hypertension were considered including renal artery stenosis, nephropathy, thyroid dysfunction, and accidental drug ingestion.

## Investigations

Transthoracic echocardiography showed unobstructed Fontan circulation, normal right ventricular function, and an unrestrictive atrial septal defect. Electrocardiogram revealed biventricular and biatrial enlargement, consistent with prior tracings.

**Figure 1:**
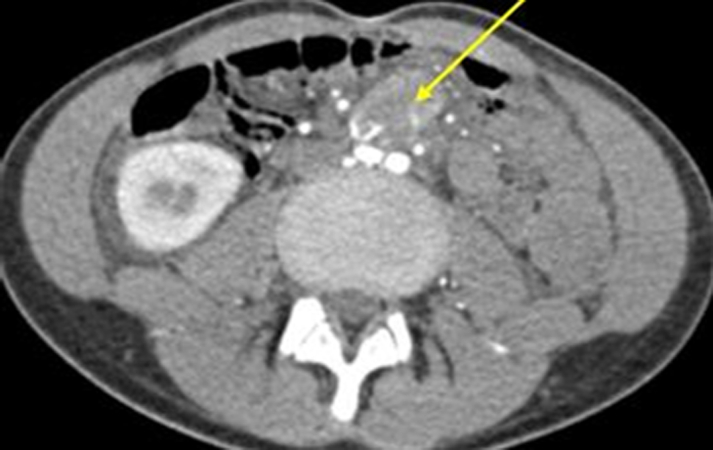
Axial Computed Tomography Angiography of the Abdomen and Pelvis Showed a Hyperenhancing Mass at the Aortic Bifurcation With Early Arterial Enhancement, Highly Suspicious for Being Malignant The yellow arrow indicates the hyperenhancing mass.

**Figure 2:**
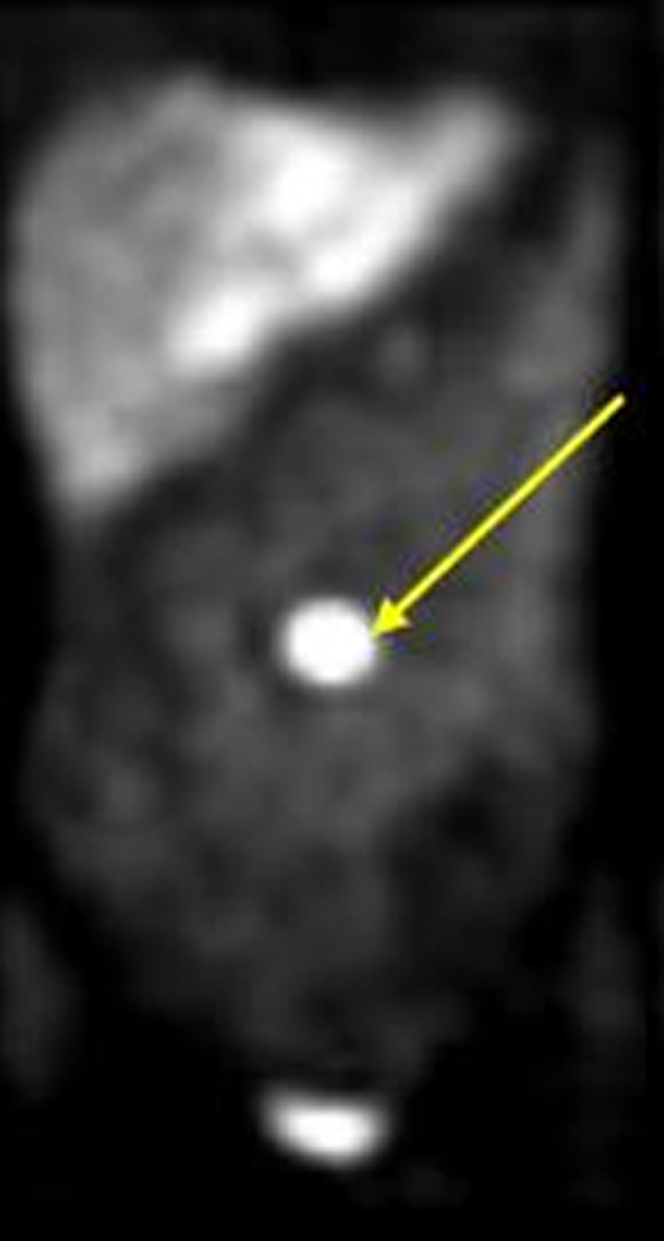
Image of the Patient's Metaiodobenzylguanidine Showing a Contrast-Avid Tumor in the Midline/Left Para Midline Abdomen Corresponding to the Hypervascular Periaortic Bifurcation Mass Without Evidence of Metastatic Disease The yellow arrow indicates the contrast-avid tumor.

Renal ultrasound revealed a small, atrophic left kidney and elevated midaortic velocities. A heterogenous, vascular mass at the aortic bifurcation was noted. Doppler ultrasound raised concern for a catecholamine-secreting tumor. Labs (Table 1) were significant for elevated urine catecholamines and metanephrines which confirmed our suspicion. Computed tomography angiography and metaiodobenzylguanidine scan localized the mass to the aortic bifurcation without metastasis (Figures 1 and 2). Genetic testing revealed variant of uncertain significance in *MYH6* and *MT-ND2*.

**Table 1:** Laboratory Results and Expected Normal Range Values for Age

Laboratory Results	Normal Range
Urine Cr.: 81.4	10-20 mg/dL
Urine protein: <5	6-12 mg/dL
Urine norepinephrine: 1,173	15-80 μg/24 h
Urine epinephrine: 5.7	0.5-20 μg/24 h
Urine dopamine: 141	65-400 μg/24 h
Normetanephrines: 14	<0.9 nmol/L
Metanephrine free: 0.53	<0.5 nmol/L
Urine vanillylmandelic acid: 12	<8 mg/g Cr.
Urine homovanillic acid: 4.3	<9 mg/g Cr.

Cr = creatinine.

## Management

Prazosin was initiated for alpha blockade; enalapril and hydrochlorothiazide were discontinued. Atenolol was added for beta blockade. She was discharged on hospital day 12 with stable blood pressure, pending elective surgical resection.

The patient was readmitted 24 hours preoperative for optimization. She underwent successful laparoscopic resection of the retroperitoneal mass. Postoperative complications included transient hypoxemic hypercapnic respiratory failure and hypotension, managed with diuretics and vasopressors. She was restarted on amlodipine and discharged on postoperative day 8.

## Outcome and Follow-Up

The patient was discharged on amlodipine, furosemide, and aspirin. She remains under the care of cardiology, nephrology, endocrinology, and her pediatrician.

## Discussion

Pheochromocytomas are rare catecholamine-producing tumors from adrenal chromaffin cells. When extra-adrenal, they are termed paragangliomas; collectively, they are called pheochromocytoma-paraganglioma (PPGL). Pediatric incidence is approximately 1 to 2 per million annually.^1^ PPGLs may present subtly—pediatric clues include paroxysmal or sustained hypertension, headache, diaphoresis, palpitations, and weight loss.^2^ Plasma-free metanephrines are the diagnostic gold standard because of their high sensitivity and specificity. Magnetic resonance imaging is the preferred imaging modality in pediatric patients; however, computed tomography remains the gold standard. Metaiodobenzylguanidine provides additional localization and staging information, particularly in high-risk or unstable cases, as in our patient.^2^

There is a recognized association between PPGL and chronic hypoxia. Hypoxia-inducible factors, especially hypoxia-inducible factor-2α, play a key role in PPGL tumorigenesis.^3^ Fontan patients—often hypoxic during early life—are predisposed. Case series report Fontan patients developing PPGL with latency periods of 10.4 to 39 years.^4^ Although our patient presented only 6 years post-Fontan, her early diagnosis underscores the need for vigilance even in younger children.

Management of PPGL is guided by Endocrine Society Guidelines,^5^ which recommend 7 to 14 days of preoperative alpha blockade to stabilize blood pressure and heart rate, followed by beta blockade if needed. Our approach adhered to these principles with prazosin and atenolol. Intraoperative and postoperative complications are common and require intensive care unit–level care because of risks of hypertensive crises, arrhythmias, and volume shifts.^5^

Although the tumor in this case was localized and operable, lifelong follow-up is essential given the potential for recurrence. In the absence of recognized familial syndromes associated with PPGL, it remains unclear how our patient's genetic variant of uncertain significance might have played a role.

## Conclusions

PPGL, although rare, should remain a high-priority consideration in pediatric patients presenting with unexplained hypertension—especially those with a history of cyanotic congenital heart disease. This case emphasizes the importance of broad differential thinking, early endocrine involvement, and multidisciplinary care in achieving safe and successful outcomes.

## Funding Support and Author Disclosures

The authors have reported that they have no relationships relevant to the contents of this paper to disclose.
